# Complete mitochondrial DNA sequence of the Japanese endemic catfish *Silurus biwaensis* (Siluriformes: Siluridae) from Lake Biwa

**DOI:** 10.1080/23802359.2021.1920487

**Published:** 2021-08-09

**Authors:** Yuu Kishimoto, Hisashi Okuyama, Jun-ichi Takahashi

**Affiliations:** Faculty of Life Sciences, Kyoto Sangyo University, Kyoto, Japan

**Keywords:** Next-generation sequencing, catfish, Lake Biwa, *Silurus biwaensis*, mitochondrial DNA

## Abstract

The Japanese endemic catfish *Silurus biwaensis* is distributed only in Lake Biwa and Yodo river drainages. There are four species of the genus *Silurus* in Japan, of which *S. biwaensis* has a most limited distribution. This catfish needs to be collected for DNA data owing to the lack of information related to its phylogenetic relationship. Here, the complete mitochondrial genome of the *S. biwaensis* from Lake Biwa in Japan was analyzed using next-generation sequencing. The mitochondrial genome of *S. biwaensis* was identified as a 16,531 bp circular molecule containing 13 protein-coding genes (PCGs), 22 tRNA genes, and 2 rRNA genes, along with one A + T-rich control region. The AT content was 55.83%. The heavy (H)-strand was predicted to have 12 PCGs, 14 tRNA, and 2 rRNA genes, whereas the light (L)-strand was predicted to contain one PCG and eight tRNA genes. The start codons ATG, ATC, and GTG were found in 13 PCGs. The stop codons TAA, TAG, and AGA were observed in all PCGs, except *CytB* and *COX3*. All tRNA genes formed typical cloverleaf secondary structures. The molecular phylogenetic relationships estimated using 13 PCGs (maximum-likelihood method) indicated that *S. biwaensis* is genetically distinct from the sympatric species *S. asotus* and *S. lithophilus*. This result clearly indicated that *S. biwaensis* is a valid species.

The Japanese endemic catfish *Silurus biwaensis* Tomoda, 1961 is distributed only in Lake Biwa and Yodo river (Maehata 2001; Tomoda 1962). *S. biwaensis* is the largest of the native fishes, attaining a length of over 1 m. This species has a most limited distribution and information on its phylogenetic position (Tabata et al. 2016; Hibino and Tabata 2018). Mitochondrial DNA sequences can estimate phylogenetic relationships. Although information on complete mitochondrial DNA sequences is abundant in several fish (Iwasaki et al. 2013), it is lacking in this species. Here, we report the complete mitochondrial genome of the *S. biwaensis* from Lake Biwa in Japan.

DNA sample from the fin of *S. biwaensis* found in Lake Biwa (35°24′N 136°08′E) was immediately extracted using the DNeasy mini kit (QIAGEN Hilden, Germany) . The specimen was stored in the Shiga Prefectural Lake Biwa Museum, Japan (Specimen number 1210058081). The gDNA library used for sequencing was prepared using the KAPA Hyper Prep kit, and a MiSeq sequencer (ILLUMINA) was used to sequence the whole genome with an Illumina reagent kit. The gDNA library was indexed and run simultaneously over 600 cycles yielding paired reads of 250 bp.

The resultant reads were assembled and annotated using the Geneious R9 (Biomatters Auckland, New Zealand) (Kearse et al. 2012) and MITOS web server (Bernt et al. 2013), respectively. Thirteen protein-coding genes (PCGs) sequences were aligned using Genetyx version 15 (GENEYTX, Tokyo, Japan). The phylogenetic analysis (maximum-likelihood analysis) was based on the nucleotide sequences of 13 PCGs using MEGA X (Kumar et al. 2018) . The general time-reversible model and gamma-distributed with invariant sites were selected from the find best DNA program in MEGA X.

We succeeded in sequencing the entire mitochondrial genome of *S. biwaensis* from Lake Biwa, Japan. This sequence was given the DDBJ accession number LC574781. The genome comprised a 16,531 bp long closed loop, including 13 PCGs, 22 tRNA genes, 2 rRNA genes, and 1 AT-rich control region, similar to the typical catfish mitochondrial genomes (Nakatani et al. 2011; Zeng et al. 2011; Vittas et al. 2011; Lian et al. 2015; Wang, Xu, Cui, et al. 2015, Wang, Xu, Xu, et al. 2015). The heavy (H)-strand was predicted to have 12 PCGs, 14 tRNA, and 2 rRNA genes, whereas the light (L)-strand was predicted to contain 1 PCGs and 8 tRNA genes. Among the 13 PCGs, the start codon ATG was found in 11, ATC and GTG in 1 gene, respectively. As a stop codon, eight, two, and one gene used TAA, TAG, and AGA. Incomplete stop codons were identified in *CytB* and *COX3*. All tRNA genes formed typical cloverleaf secondary structures.

Phylogenetic analysis was performed using the sequences of 13 mitochondrial PCGs and those of 13 closely related taxa (Figure 1). The phylogenetic analyses of the complete mitochondrial DNA genes strongly supported the result obtained from the phylogenetic analysis of partial DNA sequences, grouping the monophyletic species within the genus *Silurus*. The phylogenetic analysis also suggested that *S. biwaensis* from Lake Biwa is other taxon to sympatric species *S. asotus* and *S. lithophilus*. The results also clearly indicated that *S. biwaensis* is confirmed as a valid species.

**Figure 1.**
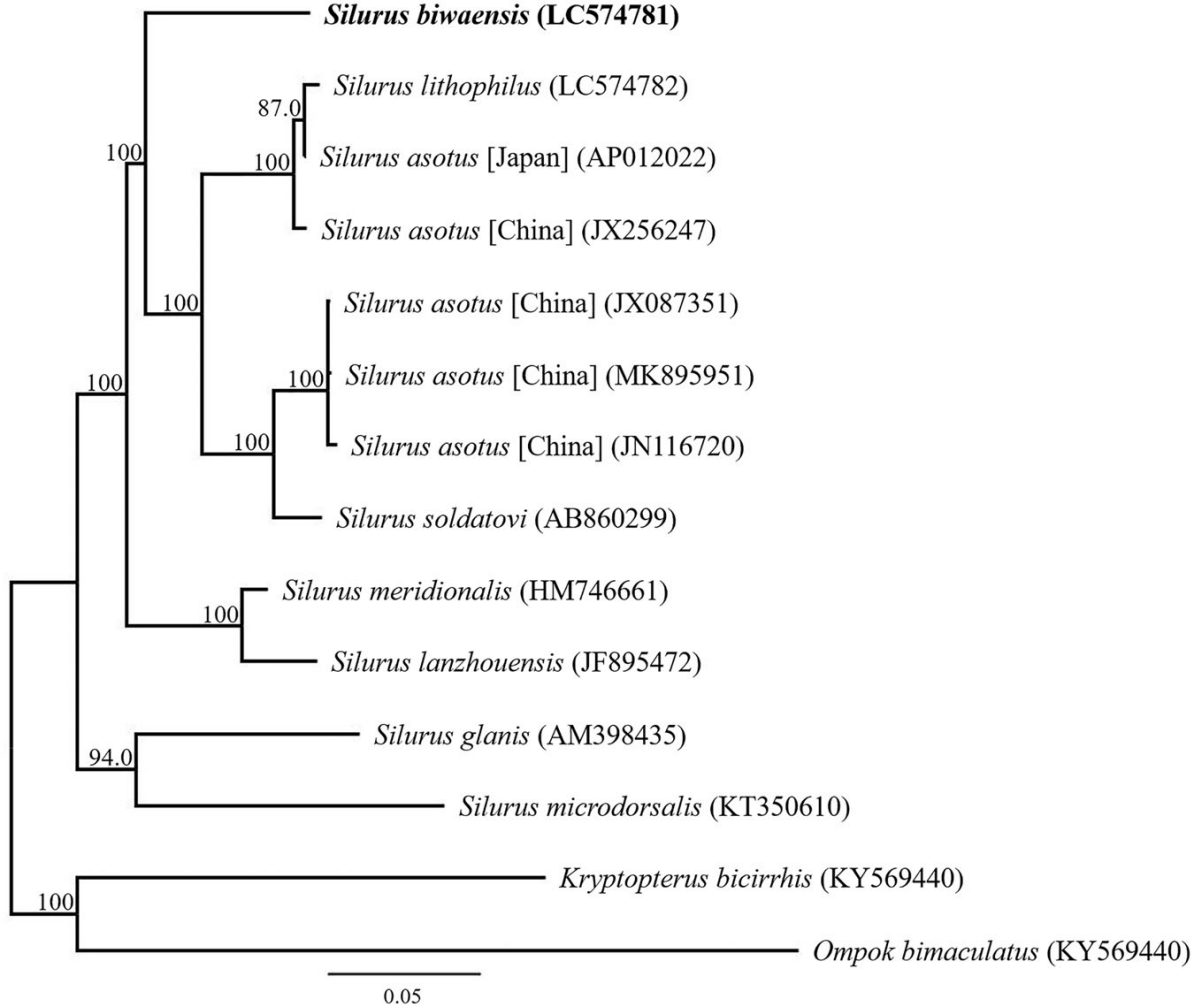
Phylogenetic relationships (maximum likelihood) of the Siluridae based on the nucleotide sequences of the 13 protein-coding genes of the mitochondrial genome. Sequences from *Kryptopterus bicirrhis* and *Ompok bimaculatus* were used as an outgroups. These sequences were separated by codon positions, and for each partition, the optimal models of sequence evolution were used in the maximum likelihood method using MEGA X, based on the corrected Akaike information criterion. The numbers at the nodes indicate the bootstrap support inferred from 1000 bootstrap replicates. Alphanumeric terms indicate the DNA Database of Japan accession numbers.

## Data Availability

The genome sequence data that support the findings of this study are openly available in DDBJ/GenBank at (https://www.ddbj.nig.ac.jp/index.html) under accession no. LC574781. The associated BioProject ID, BioSample ID, and SRA (DRA) Accession no. are PRJDB11311, SAMD00283357, and DRA011642, respectively.
